# Exosomes Derived from Glioma Cells under Hypoxia Promote Angiogenesis through Up-regulated Exosomal Connexin 43

**DOI:** 10.7150/ijms.71912

**Published:** 2022-07-04

**Authors:** Zhang-Jian Yang, Qiu-Chen Bi, Li-Jun Gan, Le-Ling Zhang, Min-Jun Wei, Tao Hong, Rong Liu, Cheng-Lin Qiu, Xiao-Jian Han, Li-Ping Jiang

**Affiliations:** 1 Jiangxi Provincial Key Laboratory of Drug Targets and Drug Screening, School of Pharmaceutical Science, Nanchang University, Nanchang 330006, China.; 2 Department of Neurosurgery, First Affiliated Hospital of Nanchang University, Nanchang 330006, China.; 3 Institute of Geriatrics, Jiangxi provincial People's Hospital, First Affiliated Hospital of Nanchang Medical College, Nanchang, 330006, China.; 4 Department of Neurology, Jiangxi provincial People's Hospital, First Affiliated Hospital of Nanchang Medical College, Nanchang, 330006, China.

**Keywords:** exosome, connexin 43, angiogenesis, hypoxia, glioma

## Abstract

Glioblastoma multiform (GBM) is a highly aggressive primary brain tumor. Exosomes derived from glioma cells under a hypoxic microenvironment play an important role in tumor biology including metastasis, angiogenesis and chemoresistance. However, the underlying mechanisms remain to be elucidated. In this study, we aimed to explore the role of connexin 43 on exosomal uptake and angiogenesis in glioma under hypoxia. U251 cells were exposed to 3% oxygen to achieve hypoxia, and the expression levels of HIF-1α and Cx43, involved in the colony formation and proliferation of cells were assessed. Exosomes were isolated by differential velocity centrifugation from U251 cells under normoxia and hypoxia (Nor-Exos and Hypo-Exos), respectively. Immunofluorescence staining, along with assays for CCK-8, tube formation and wound healing along with a transwell assay were conducted to profile exosomal uptake, proliferation, tube formation, migration and invasion of HUVECs, respectively. Our results revealed that Hypoxia significantly up-regulated the expression of HIF-1α in U251 cells as well as promoting proliferation and colony number. Hypoxia also increased the level of Cx43 in U251 cells and in the exosomes secreted. The uptake of Dio-stained Hypo-Exos by HUVECs was greater than that of Nor-Exos, and inhibition of Cx43 by ^37,43^gap27 or lenti-Cx43-shRNA efficiently prevented the uptake of Hypo-Exos by recipient endothelial cells. In addition, the proliferation and total loops of HUVECs were remarkably increased at 24 h, 48 h, and 10 h after Hypo-Exos, respectively. Notably, ^37,43^gap27, a specific Cx-mimetic peptide blocker of Cx37 and Cx43, efficiently alleviated Hypo-Exos-induced proliferation and tube formation by HUVECs. Finally, ^37,43^gap27 also significantly attenuated Hypo-Exos-induced migration and invasion of HUVECs. These findings demonstrate that exosomal Cx43 contributes to glioma angiogenesis mediated by Hypo-Exos, and suggests that exosomal Cx43 might serve as a potential therapeutic target for glioblastoma.

## Introduction

Glioma is one of the most common malignant brain tumors, accounting for more than 45% of total tumors in the central nervous system. The prognosis of glioma is always poor, with patients suffering from glioma having a median overall survival of only 14.6 months 1. Conventional therapeutic strategies include surgery, radiotherapy, chemotherapy or multimodality therapy 2. Unfortunately, the current therapeutic strategies do not significantly improve the prognosis of glioma. For solid tumor, the tumor microenvironment, such as hypoxia, plays a pivotal role in the migration and invasion of glioma cells 3, 4. Under hypoxia, a variety of factors including HIF-1α are released to promote the migration and invasion of cancer cells, and stimulate angiogenesis, an important pathological feature of tumor progression 5, 6. However, the intercellular communication between cancer cells and vascular endothelial cells and its role in tumor progression remain to be elucidated.

Under a hypoxic tumor microenvironment, there are proximal and long-distance signaling between cancer cells and vascular endothelial cells to regulate neovascularization 7. Most previous studies focus on the paracrine signaling pathway mediated by vascular endothelial growth factor (VEGF), however, emerging evidence indicates that exosomes play a vital role in the long-distance signaling in a hypoxic tumor microenvironment 8. Exosomes (Exos) are composed of double concave vesicles with a diameter ranging from 30 to 150 nm, which can mediate long-distance intercellular signal transduction and transportation of substances 9. Several studies have demonstrated that cancer cell-derived Exos stimulate neovascularization, regulate tumor immune microenvironment as well as tumor metastasis 10-12. The soluble E-cadherin on exosomal membranes can form heterodimers with VE-cadherin of endothelial cells, promoting tumor angiogenesis via activation of β-catenin and NF-κB signaling pathways 13. Similarly, esophageal squamous cell carcinoma-derived Exos under hypoxia also contribute to the proliferation, migration, invasion and tube formation of human umbilical vein endothelial cells (HUVECs) 14. In addition, it has been reported that hypoxic glioblastoma-derived exosomes disrupt the permeability of blood-brain barrier 15. Lang et al 16 found that Exos-derived from U87 cells promoted the migration, proliferation, and tubular-like structure formation in HUVECs via linc-CCAT2. Thus, exosomes derived from cancer cells play an important role in angiogenesis in a tumor microenvironment.

At present, most studies of exosomes are focused on the delivery of encapsulated signaling molecules including proteins, microRNAs, long noncoding RNAs, *etc*, and their effects on cellular activities 17. However, the function of exosomal membrane components is still unclear. Although exosomal membranes originate from plasma membranes, exosomal membranes may contain some unique components 18. It has also been reported that exosomal membrane components may affect exosome uptake by target cells 19. It is well known that connexins (Cxs) are a family of transmembrane proteins that assembles to form gap junctions and connect the cytoplasm of adjacent cells for intercellular communication 20. Recently, the abnormal expression of Cx43 in tumor was found to be closely related to cancer recurrence and metastasis 21-24. Cx26, Cx43 and Cx45 were detected in 35 cases of oral squamous cell carcinoma, and the high level of Cx43 was related to the short overall survival period of patients 25. The expression level of Cx43 was also inversely correlated with the survival rate of patients suffering esophageal squamous cell carcinoma 26. Furthermore, it has also been reported that Cx43 can regulate the interaction and communication between Exos and cells 27. However, whether the tumor hypoxic microenvironment stimulates angiogenesis through exosomal Cx43-mediated signaling remains to be further investigated.

In this study, the exosomes were isolated from glioma U251 cells under hypoxia or normoxia, respectively. It was found that Cx43 was up-regulated in U251 cell-derived exosomes under hypoxia (Hypo-Exos) and that the uptake of Hypo-Exos by HUVECs was greater than that of Nor-Exos. Notably, Cx43 inhibitor ^37,43^gap27 or knockdown of Cx43 by lenti-Cx43-shRNA efficiently prevented the exosomal uptake by HUVECs. Moreover, Hypo-Exos significantly increased the proliferation, tube formation and migration of HUVECs, while ^37,43^gap27 alleviated the Hypo-Exos-induction of proliferation, migration and angiogenesis in the HUVECs. All these results suggest the important role of Cx43 in exosomal uptake and angiogenesis under hypoxia, and provide the exosomal Cx43 as a potential therapeutic target for glioblastoma.

## Materials and Methods

### Reagents

Primary antibody against HIF-1α and HRP-conjugated secondary antibodies were purchased from Cell Signaling Technology®, Inc. (Boston, MA, USA). Primary antibodies against CD63, TSG101 and β-actin were obtained from Affinity Biosciences (OH, USA), and primary antibody against Cx43 was from Abcam (Cambridge, UK). 37,43gap27 was obtained from Eurogentec (Liege, Belgium). Transwell chamber were purchased from Corning, Inc. (NY, USA), and Giemsa was obtained from Merck, Co., Ltd. (Darmstadt, Germany).

### Cell lines and cell culture

Human glioblastoma cell line U251 was obtained from the American Type Culture Collection (ATCC), and the human umbilical vein endothelial cell line (HUVECs) was obtained from the Cell Resource Center of Shanghai Institutes for Biological Sciences, Chinese Academy of Sciences. The two kinds of cells were grown at 37℃ in high glucose DMEM medium (Solarbio, Beijing, China) supplemented with 10% fetal bovine serum (Biotech Co., China) and 1% penicillin/streptomycin (Solarbio, Beijing, China) in a humidified incubator with an atmosphere containing 5% CO2. To achieve hypoxia, U251 cells were incubated with mixed air containing 3% O2, 5% CO2 and 92% N2.

### Western Blotting assay

Expression levels of the proteins of interest were examined by Western Blot analysis as previously described 28. Briefly, cells or the isolated exosomes were harvested and lysed in radioimmunoprecipitation assay (RIPA) buffer (Solarbio, Beijing) with protease inhibitors. Proteins were run on 10% SDS-PAGE gels, and then transferred to PVDF membrane (Millipore, MA, USA). After blocking in 5% skim milk for 1 h at room temperature, membranes were immunoblotted with primary antibodies against HIF-1α, TSG101, CD63, Cx43 (1:1,000) or β-actin (1:2,000) at 4 ℃ overnight. After washing three times with TBST, the membranes were further incubated with HRP-conjugated secondary antibodies (1:3,000) for 2 h at room temperature. Chemiluminescence signals were detected using the ECL-detecting reagents (CWBIO, Beijing, China). Densitometric analysis was conducted with the Gel Imaging System (Analytik, Jena, Germany).

### Colony formation assay

To evaluate the effect of hypoxia on colony formation of glioma cells, U251 cells were exposed to normoxia or hypoxia (3% O2) for 48 h. Cells were harvested and resuspended at a density of 6×103/mL. 50 μl of cell suspension was plated on a 6-well plate, and cultured for a further 10 days. Then, cells were washed with PBS and fixed in 4% paraformaldehyde (PFA) (Sigma‑Aldrich) for 30 min on ice. 2 mL of Giemsa (Merck, Co., Ltd., Germany) was added to each well and cells were stained for 20 min. After washing 3 times with PBS, colonies with more than 40 cells were counted.

### CCK-8 assay

The proliferation of U251 cells was evaluated using a CCK-8 assay according to the manufacturer's instructions. In brief, cells were separately seeded into 96-well plates at approximately 2×103 cells/well and cultured in complete growth medium. To examine the effect of hypoxia on cellular proliferation, U251 cells were maintained under normoxia or hypoxia for 24 h, 48 h, respectively. In addition, the effect of normoxia- or hypoxia-derived exosomes on the proliferation of U251 cells was evaluated. Cells were treated with PBS (control), Nor-Exos (50 μg/ml), Hypo-Exos (50 μg/ml) or 37,43gap27 (100 μg/ml) +Hypo-Exos for 24 h, or 48 h, respectively. Then, cells were incubated with 10 μL of CCK-8 reagent (Vazyme biotech co., Ild., Nanjing, China) at 37 ℃ for 2 h. The absorbance at 450 nm was measured using a multifunctional microplate reader (Bio-Rad, USA) to evaluate cellular proliferation. Three replicate wells were implemented in each group, and all data were from three independent experiments.

### Isolation and identification of exosomes

For the isolation of exosomes, U251 cells were first cultured in serum-free medium for 48 h. Then, the culture medium was collected and subjected to differential centrifugation using sequential centrifugations of 300 g for 10 min, 2000 g for 10 min, 10,000 g for 30 min and 100,000 g for 70 min at 4℃. The exosomes were harvested from the pellet and resuspended in 1×PBS. To identify the isolated exosomes, the particle size, morphology and protein marker TSG101 and CD63 of exosomes were assessed using the Zetasizer Nano ZS 90 particle size analyzer (Malvern Panalytical, UK), transmission electron microscopy (Leica, Germany) and Western blotting assay as previously described 26.

### Detection of exosome uptake

To label exosomes, the isolated exosomes were incubated with 10 μM of dioctadecyloxacarbocyanine (Dio, Beyotime Biotechnology Co., Ltd, Shanghai, China) at 37 ℃ for 30 min according to the manufacturer's instructions. The Dio-labeled exosomes were resuspended with sterile 1×PBS and subjected to centrifugation at 100,000 g to remove the free Dio. To detect exosome uptake, HUVECs cells were treated with 50 μg/mL of Dio-labeled Nor-Exos or Hypo-Exos for 30 min in darkness. Then, cells were washed twice with 1×PBS, and fixed with 4% PFA for 10 min on ice. The nuclei of HUVECs were counterstained with 500 μL of Hoechst 33342 (10 μg/ml) (Beyotime Biotechnology Co., Ltd, Shanghai, China) for 5 min in darkness. The uptake of Dio-labeled exosomes by HUVECs was detected under an inverted fluorescence microscope (Leica, Germany).

### Tube formation assay

HUVECs (2×104 cells/well) were seeded on a 48-well plate coated with growth factor-reduced Matrigel (Corning, Inc., NY, USA) and incubated with PBS, Nor-Exos, Hypo-Exos or 37,43gap27 (100 μg/ml) + Hypo-Exos for 10 h. The tube formation was observed and photographed under an inverted microscope. The number of total loops in HUVECs was quantified from images of five random fields under the microscopy using Image-Pro Plus 6.0 software. Each experiment was repeated three times.

### Wound healing and Transwell assay

The wound healing and Transwell assay were conducted as described previously 29. In brief, the confluent monolayer of HUVECs was mechanically wounded by 200 μL pipette tip, and the suspended cells were removed. Then, HUVECs were incubated with PBS, Nor-Exos, Hypo-Exos or Hypo-Exos+^37,43^gap27, respectively. The wound healing was photographed under an inverted fluorescence microscope at 0 h, 24 h or 48 h after the above treatments, and the wound closure was measured using image J software (NIH, Bethesda, MD, USA) to evaluate migration of HUVECs. In addition, transwell chambers (Corning, Inc., NY, USA) were used for transwell assays. 3×10^5^ HUVECs were seeded into the upper chamber containing 200 μL serum-free DMEM medium supplemented with PBS, Nor-Exos, Hypo-Exos or Hypo-Exos+^37,43^gap27. 500 μL complete medium with 10% FBS was added into the lower chamber. The migrated cells on the surface of the lower chamber at 24 h or 48 h were fixed with 4% paraformaldehyde for 10 min and stained with 0.1% crystal violet for 30 min. Then, migrated cells were photographed under an inverted microscope and counted in four random fields.

### Establishment of Cx43 Knockdown U251 stable cell line

For knockdown of Cx43 in U251 cells, shRNAs targeting Cx43 and its scramble sequence (negative control) were subcloned into the lentiviral vector p-FU-GW-007 carrying a green fluorescent protein by Genechem Co.,Ltd (Shanghai, China). The sequence of three shRNAs targeting Cx43 and the scrambled control are presented in table 1. First, the concentration of puromycin for screening Cx43 knockdown stable cell lines was determined as previously describes 30. Briefly, U251 cells at a density of 1.2×103 cells/well were seeded on 24-well plates. 24 h later cells were further cultured in growth medium containing 0, 0.25, 0.5, 1.0, 1.25 and 1.5 µg/ml of puromycin for 7 days. U251 cell survival state was monitored every 24 h under a bright-field microscope. The minimum concentration of puromycin needed to induce complete cell death was determined as the screening concentration of puromycin for U251 cells. To establish the Cx43 knockdown stable cell line, U251 cells were seeded on 6-well plates at a density of 3×105 cells/well and infected with Lenti-Cx43 shRNAs or scrambled control. At 48 h after infection, U251 cells were selected by puromycin at screening concentration. Then, the selected colonies with GFP fluorescence were picked and passaged. The Cx43 knockdown U251 stable cell line were confirmed by fluorescence microscopy and western blotting.

### Statistical analysis

Data were presented as the mean ± SEM. The comparison between two groups was analyzed by unpaired two-tailed Student's t-test. The comparison among multiple samples were performed by a one-way ANOVA parametric test followed by Tukey's multiple comparisons test. P<0.05 was considered statistically significant. The experimental data were analyzed using the GraphPad 5.0 software.

## Results

### Hypoxia up-regulated HIF-1α expression and promoted the colony formation and proliferation of U251 cells

**Figure 1:**
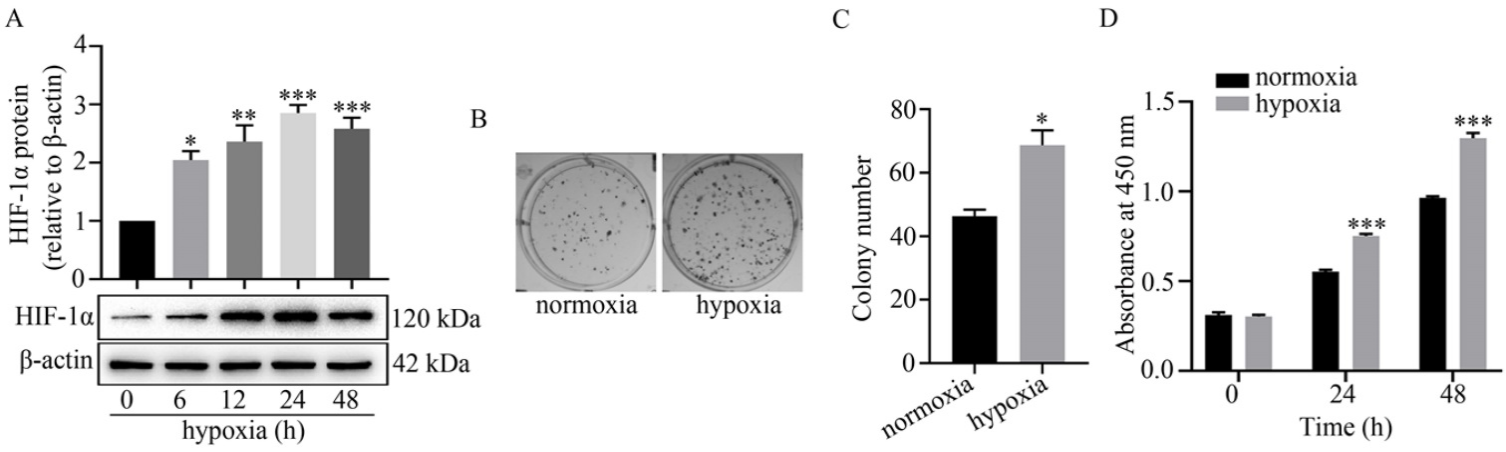
** Effect of hypoxia on HIF-1α expression, colony formation and proliferation of U251 cells.** (A) Effect of hypoxia on HIF-1α expression in U251 cells at the indicated time points as indicated by the relative expression levels of HIF-1α in U251 cells. The relative level of HIF-1α/β-actin in each group was normalized to that at 0 h. ^*^P<0.05, ^**^P<0.01 and ^***^P<0.001 *vs* 0 h. (B) Representative images of colony formation of U251 cells under normoxia or hypoxia. (C) Displays the colony numbers observed in B represented numerically by bar graph. ^*^P<0.05 *vs* normoxia. (D) Represents the proliferation of U251 cells under normoxia or hypoxia at 0 h, 24 h, 48 h as detected by CCK-8 assay. All data presented represents at least three independent experiments. ^***^P<0.001 *vs* normoxia.

It is well known that hypoxia regulates cellular activities by stabilizing HIF-1α 3. Here, the expression level of HIF-1α was detected by western blot analysis to determine the successful establishment of a hypoxic model in U251 cells. As shown in **Figure 1A**, HIF-1α was significantly up-regulated at 6 h to 48 h under hypoxia. In addition, hypoxia facilitated colony formation in U251 cells. Compared to normoxia (45±5), the colony number under hypoxia (73±6) was markedly increased (**Figure 1B-C**). The results of the CCK-8 assay also revealed that proliferation of U251 cells under hypoxia at 24 h and 48 h was significantly greater than that under normoxia (**Figure 1D**).

### Isolation and identification of Exos from U251 cells under normoxia and hypoxia

**Figure 2:**
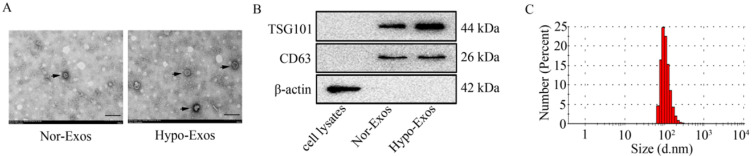
**Identification of exosomes isolated from U251 cells under normoxia or hypoxia.** (A) The representative images of Nor-Exos and Hypo-Exos (black arrows) from U251 cells were captured by transmission electron microscopy (×40,000). Scale bar, 200 nm. (B) The exosomal markers TSG101 and CD63 are expressed in whole cell lysates, Nor-Exos and Hypo-Exos. (C) The diameters of Nor-Exos and Hypo-Exos measured by the Zetasizer Nano ZS90 particle size analyzer show particles in the correct size range for exosomes.

Next, Exos were isolated from U251 cells under normoxia or hypoxia at 48 h by differential velocity centrifugation according to the previous study 31. The morphology of isolated vesicles was assessed by transmission electron microscopy (as shown in **Figure 2A**). To identify the isolated vesicles, the expression of exosomal markers including TSG101 and CD63 was examined by Western blot analysis. Results showed that both exosomal markers, CD63 and TSG101, were detected in isolated Nor-Exos or Hypo-Exos (**Figure 2B**). Similar to previously reported sizes of exosomes (30~150 nm in diameter) 32, the size of isolated vesicles from U251 cells was in the range of 80~130 nm (**Figure 2C**). These data indicate that the Exos (Nor-Exos and Hypo-Exos) are successfully isolated from U251 cells under normoxia and hypoxia, respectively.

### Hypoxia up-regulated the expression of Cx43 in the isolated Exos from U251 cells

**Figure 3:**
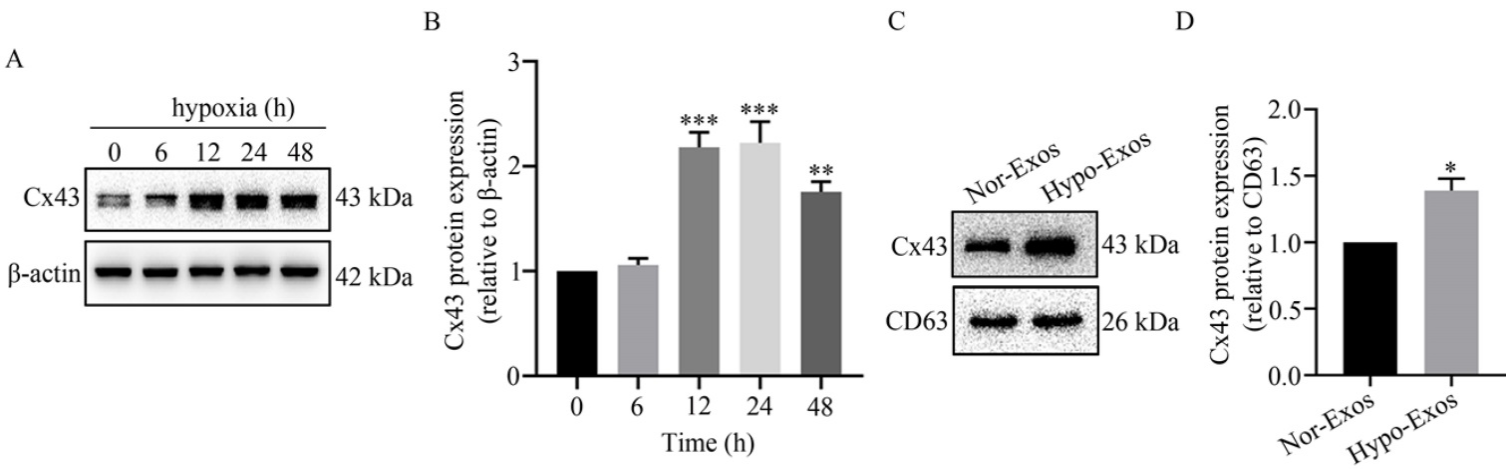
**Effect of hypoxia on Cx43 expression in U251 cells and their exosomes.** (A) The expression of Cx43 in U251 cells under hypoxia for 0~48 h. β-actin was used as an endogenous control. (B) The relative expression level of Cx43 in U251 cells under hypoxia at different time points. The relative level of Cx43/β-actin in each group was normalized to that at 0 h. ^**^P<0.01 and ^***^P<0.001 *vs* 0 h. (C) Expression of Cx43 in Nor-Exos and Hypo-Exos at 48 h. CD63 expression was used as the endogenous reference. (D) The relative expression levels of Cx43 in Nor-Exos and Hypo-Exos was quantified. ^*^P<0.05 *vs* Nor-Exos.

Previous reports in the literature point to the ability to transfer microRNAs from donor glioma cells to neighboring cells via Cx43 33. To examine the role of Cx43 in exosomal signaling transduction, the expression of Cx43 in U251 cells and the isolated Exos was detected using Western blot analysis. As shown in** Figure 3A and 3B**, Cx43 was significantly up-regulated in U251 cells at 12 h to 48 h under hypoxia. Similarly, the expression level of Cx43 in Hypo-Exos was significantly higher than that in Nor-Exos (**Figure 3C and 3D**). These results suggest that hypoxia up-regulates the gap junction protein Cx43 in isolated Exos from glioma U251 cells. However, the role of Cx43 in exosomal signaling transduction remains to be elucidated.

### Cx43 promoted the proliferation and angiogenesis of HUVECs

**Figure 4:**
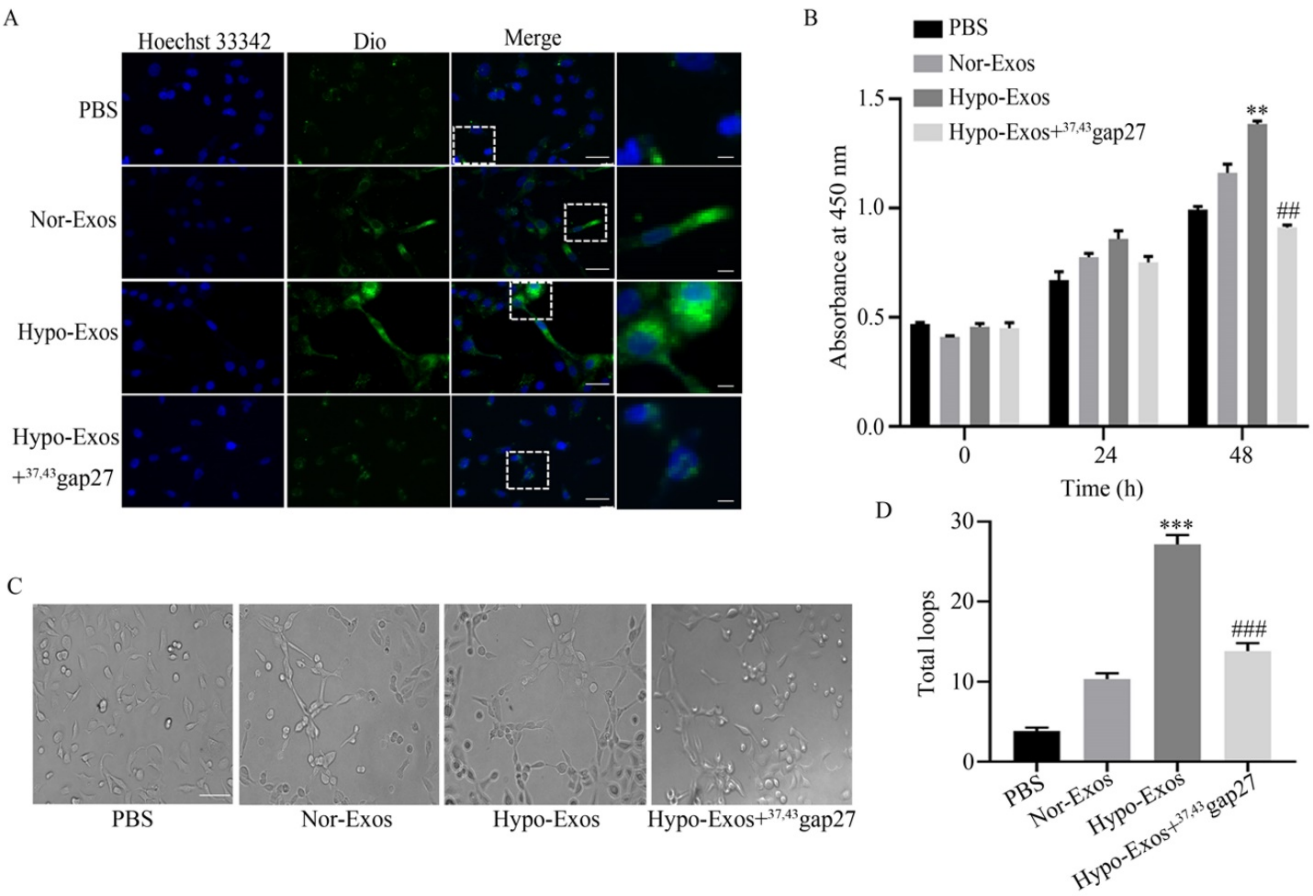
**The role of Cx43 on exosomal uptake, proliferation and tube formation in HUVECs.** (A) The uptake of Dio-Nor-Exos and Dio-Hypo-Exos by HUVECs. Cells were incubated with PBS, Dio-Nor-Exos, Dio-Hypo-Exos or Dio-Hypo-Exos+^37, 43^Gap27 and counterstained with Hoechst 33342 (blue). Dio-stained exosomes (green) in cells was detected using fluorescence microscopy. Short Scale bar, 5 μM; Long Scale bar, 25 μM. The rightmost panels indicate enlarged images of the white dashed boxes. (B) The proliferation of HUVECs in each group treated with PBS, Nor-Exos, Hypo-Exos or Hypo-Exos+^37, 43^Gap27 at 0 h, 24 h, 48 h. Representative data of at least three independent experiments. ^**^P<0.01 *vs* Nor-Exos (48 h), ^##^*P*<0.01 *vs* Hypo-Exos (48 h). (C) Representative images of tube formation in HUVECs treated with PBS, Nor-Exos, Hypo-Exos or Hypo-Exos+^37, 43^Gap27 for 10 h. Scale bar, 100 µm. (D) The number of total loops in HUVECs treated with PBS, Nor-Exos, Hypo-Exos or Hypo-Exos+^37, 43^Gap27. ^***^P<0.001 *vs* Nor-Exos, ^###^*P*<0.001 *vs* Hypo-Exos.

It was reported that the uptake of neck squamous cell carcinoma (HNSCC)-derived Exos promoted the angiogenesis of HUVECs 34. Therefore, we explored the role of hypoxia on the uptake of U251 cell-derived Exos by HUVECs. HUVECs were incubated with PBS, Nor-Exos, Hypo-Exos or Hypo-Exos+^37,43^Gap27. The fluorescence intensity treated with Hypo-Exos in HUVECs was stronger than that treated with Nor-Exos, however, ^37,43^Gap27 attenuated these effects (**Figure 4A**). To examine the role of exosomal Cx43 in the proliferation and angiogenesis of endothelial cells, HUVECs were incubated with PBS, Nor-Exos, Hypo-Exos or Hypo-Exos+^37,43^Gap27. As shown in** Figure 4B**, Hypo-Exos significantly increased the proliferation of HUVECs at 48 h, which was reversed by Cx43 specific blocker ^37,43^gap27. Next, the role of exosomal Cx43 in angiogenesis of HUVECs was further examined. Compared with PBS and Nor-Exos, Hypo-Exos remarkedly promoted the tube formation of HUVECs at 10 h. Pretreatment with Cx43 inhibitor ^37,43^Gap27 also alleviated Hypo-Exos-induced tube formation in HUVECs (**Figure 4C and 4D**). All these results suggest that exosomal Cx43 contributes to the proliferation and angiogenesis of HUVECs.

### Exosomal Cx43 contributed to the migration and invasion of HUVECs

**Figure 5:**
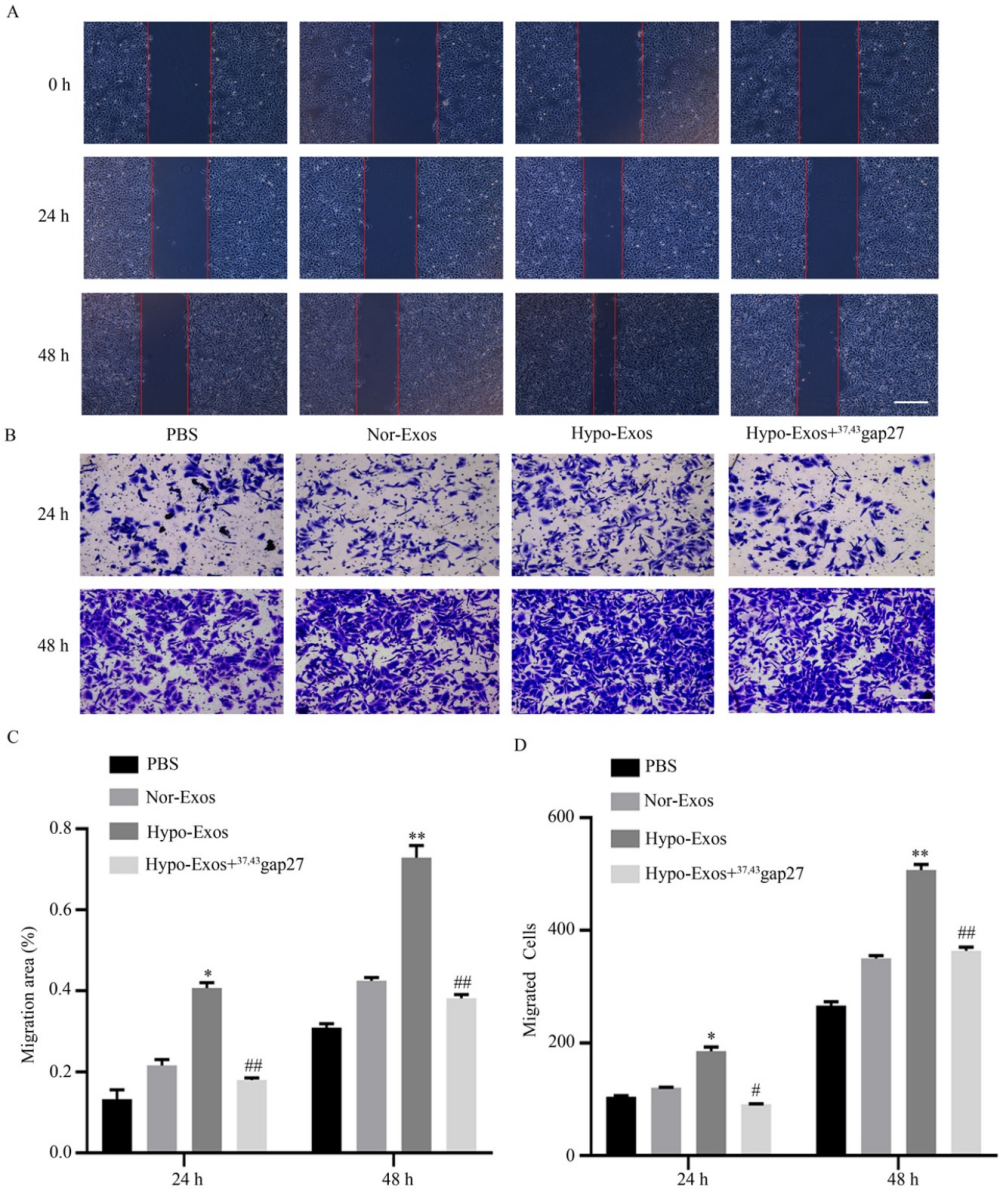
**The role of Cx43 on Hypo-Exos induced migration of HUVECs.** (A, C) The migration of HUVECs treated with PBS, Nor-Exos, Hypo-Exos or Hypo-Exos+^37, 43^Gap27 was detected using a wound healing assay at the indicated time points (A). Scale bar, 500 µm. Cell migration was quantified as the wound closure in three independent experiments for each group. ^*^P<0.05, ^**^P<0.01 *vs* Nor-Exos at the same time point, ^##^*P*<0.01 *vs* Hypo-Exos at the same time point. (B, D) The migratory activity of HUVECs treated with PBS, Nor-Exos, Hypo-Exos or Hypo-Exos+^37, 43^Gap27 for 24 h and 48 h was evaluated by Transwell assay. The migrated cells were visualized by microscopy (B). Scale bar, 200 µm. Cell migration was quantified by mean cell counts from at least 9 fields in three independent experiments for each condition (D). ^*^P<0.05, ^**^P<0.01 *vs* Nor-Exos; ^#^*P*<0.05, ^##^*P*<0.01 *vs* Hypo-Exos at the same time point.

To examine the role of exosomal Cx43 in endothelial activities, migration and invasion of HUVECs incubated with PBS, Nor-Exos, Hypo-Exos or Hypo-Exos+^37, 43^Gap27 were evaluated by wound healing and Transwell assays, respectively. As shown in **Figure 5A-D**, Hypo-Exos significantly promoted the migration and invasion of HUVECs at 24 h and 48 h. In contrast, pretreatment with Cx43 inhibitor ^37, 43^Gap27 effectively attenuated Hypo-Exos-induced migration and invasion in HUVECs. These results suggest the important role of exosomal Cx43 in the migration and invasion of endothelial cells.

### Establishment of the U251 cell lines stably expressing Cx43-shRNA

**Figure 6:**
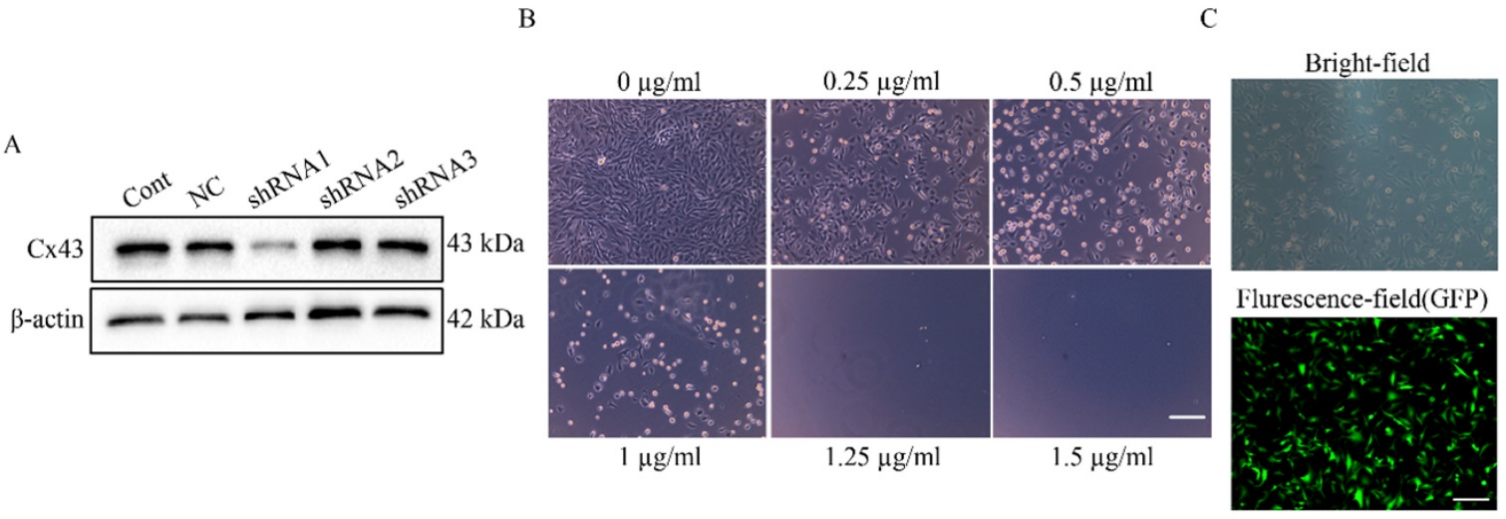
**Establishment of a U251 Cx43 knockdown stable cell line.** (A) The knockdown effect of different lenti-Cx43-shRNAs on Cx43 in U251 cells. β-actin was used as the endogenous reference. (B) Determination of the screening concentration of puromycin in U251 cells. U251 cells were incubated with 0~1.5 µg/ml of puromycin for 7 days and the minimum concentration of puromycin induced complete cell death was used as the screening concentration of puromycin. Scale bar, 200 µm. (C) The Cx43 knockdown U251 stable cell line with GFP fluorescence was selected by puromycin. The upper panel is the image of cells in bright-field, and the lower panel is the fluorescence image. Scale bar, 200 µm.

To explore the direct involvement of Cx43 in uptake of U251 cell-derived exosomes by HUVECs, we established U251 cell lines stably expressing Cx43-shRNA. First, the minimum lethal concentration of puromycin to U251 cell line was determined as 1.25 µg/ml (**Figure 6B**), and 1.5 µg/ml of puromycin was used for screening U251 stable cell lines. In transient knockdown experiments, only shRNA1 stably reduced Cx43 expression in U251 cells (**Figure 6A**). Thus, Lenti-Cx43-shRNA1 was used for establishment of Cx43-knockdown U251 cell line. In addition, analysis by fluorescence microscopy showed that U251 cell lines stably express Cx43-shRNA (**Figure 6C**).

### Exosomal Cx43 facilitated the uptake of U251 cell-derived exosomes by HUVECs

**Figure 7:**
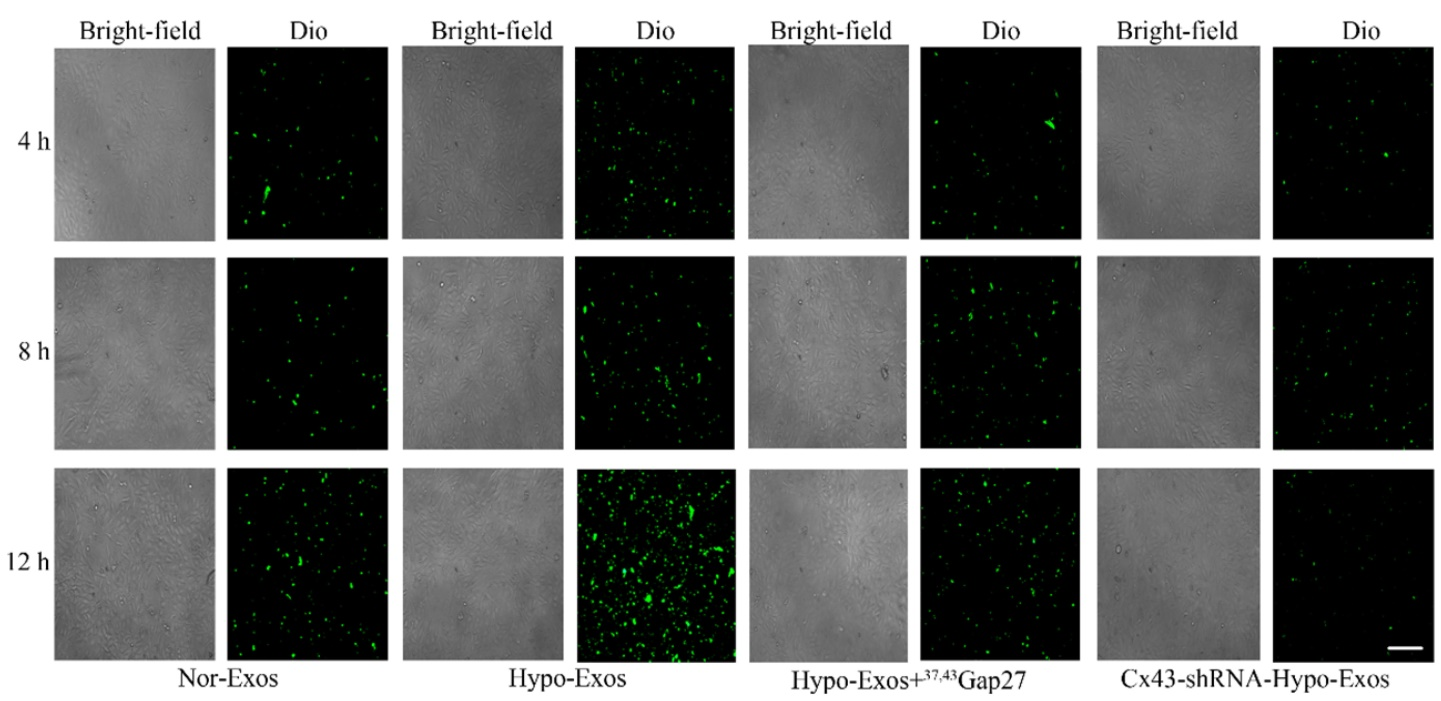
** The exosomal Cx43 mediated uptake of exosomes by HUVECs.** HUVECs were incubated with Dio-Nor-Exos, Dio-Hypo-Exos, Dio-Hypo-Exos+^37, 43^Gap27 or Cx43-shRNA-Hypo-Exos for 4 h, 8 h and 12 h. The uptake of Dio-stained exosomes (green) by cells was detected using an inverted fluorescence microscope. Scale bar, 200 µm.

The role of Cx43 in the uptake of U251 cell-derived exosomes by HUVECs was examined by incubating HUVECs with PBS, Dio-Nor-Exos or Dio-Hypo-Exos. The fluorescence of Dio in HUVECs indicates the uptake of exosomes. The results showed that the fluorescence intensity of Dio in cells treated with Dio-Hypo-Exos in HUVECs was stronger than that in cells treated with Dio-Nor-Exos (**Figure 4A**). To further investigate whether exosomal Cx43 directly affects the Exos uptake by HUVECs, HUVECs were incubated with Dio-Nor-Exos, Dio-Hypo-Exos, Dio-Hypo-Exos+^37, 43^Gap27 or Dio-Cx43-shRNA-Hypo-Exos, respectively. As shown in **Figure 7**, the fluorescence intensity of Dio in HUVECs incubated with Dio-Hypo-Exos was stronger than that in HUVECs treated with Dio-Nor-Exos groups at 8 h and 12 h. However, Cx43 inhibitor ^37,43^Gap27 or Dio-Cx43-shRNA-Hypo-Exos efficiently alleviated the uptake of exosomes by HUVECs. These results suggest that exosomal Cx43 plays an important role in the uptake of U251 cells-derived exosomes by endothelial cells.

## Discussions

In solid tumors, the uncontrolled proliferation of cancer cells usually leads to a relative lack of oxygen and nutrition, which is also termed as a hypoxic tumor microenvironment. Hypoxia regulates several activities of cancer cells via activation of a vital transcription factor HIF-1α 35 and it has been well documented that a hypoxic microenvironment contributes to the development of metastasis and chemotherapeutic resistance 4, 36. In the present study, we found that the expression of HIF-1α in glioma U251 cells was significantly up-regulated under hypoxia (Fig. 1A). Previous studies have demonstrated that mild hypoxia is beneficial to the proliferation of neural stem cells and differentiation of neurons and oligodendrocytes 37 with hypoxia-induced lncRNA LUCAT1 facilitating the growth of colorectal cancer cells 38. In accordance with previous studies, we found that hypoxia also significantly increased the colony formation and proliferation of U251 cells (Fig. 1B-C and Fig. 1D). Recently, exosomes (Exos) have been identified as the crucial cellular components for long-distance intercellular signaling transduction 39. However, the role of glioma cell-derived exosomes under hypoxia in tumor angiogenesis remains to be further investigated.

The emerging evidence has revealed that cancer cells can act as the active exosome producer, and exosomes play a vital role in tumorigenesis and development 40-42. As a unique delivery carrier, exosomes participate in the intercellular delivery of some bioactive molecules including functional proteins, noncoding RNAs and mRNAs 43. Recently, the contribution of exosomal membrane components to exosomal uptake by target cells has attracted the attention of researchers. Connexins are the vital plasma components, which are assembled into gap junctions for intercellular communication 44 with Cx43 being the most common connexin expressed in glioma cell lines 45 and the high expression level of Cx43 being reported as associated with the development of temozolomide resistance in glioma cells 46. Moreover, Joanna Gemel 47 found that the exosomal Cx43 might be involved in the docking/fusion of exosomes with recipient cells. Consistently, we found that hypoxia up-regulated Cx43 level in U251 cells-derived exosomes, and that Cx43 facilitated exosomal uptake by HUVECs (Fig. 3C, and Fig. 7). These results suggest the important role of Cx43 in the intercellular communication mediated by exosomes.

In the present study, exosomes were isolated from U251 cells under normoxia and hypoxia, respectively. It was found that the level of Cx43 in Hypo-Exos was significantly higher than that in Nor-Exos (Fig. 3C), which is interesting given that exosomes need to dock and fuse with recipient cells prior to internalization. Next, the uptake of Hypo-Exos or Nor-Exos by HUVECs was examined. Results showed that more Hypo-Exos were internalized into HUVECs, and inhibition of Cx43 by ^37,43^gap27 or lenti-Cx43-shRNA effectively prevented the uptake of Hypo-Exos by endothelial cells (Fig. 4A and Fig. 7). In previous studies, exosomes derived from esophageal squamous cell carcinoma under hypoxia were shown to promote the proliferation, migration, invasion and tube formation of HUVECs 14. Exosomes secreted by colorectal cancer cells under hypoxia was also found to facilitate the proliferation and migration of endothelial cells through the Wnt4/β-catenin signaling pathway 48. Thus, the role of exosomal Cx43 on proliferation, tube formation, migration and invasion of HUVECs was further investigated in this study. Notably, Hypo-Exos significantly increased the proliferation of HUVECs at 48 h, which was inhibited by ^37,43^gap27 (Fig. 4B). It has been well documented that hypoxic tumor microenvironment contributes to tumor growth and metastasis through angiogenesis and immune regulation 49. Here, the effect of exosomal Cx43 on angiogenesis in glioblastoma was further examined. As expected, Hypo-Exos markedly promoted the tube formation of HUVECs at 10 h, and ^37, 43^gap27 efficiently attenuated Hypo-Exos-induced tube formation of HUVECs (Fig. 4C). In addition, ^37,43^gap27 also alleviated Hypo-Exos-induced migration and invasion of HUVECs (Fig. 5A-D). This study demonstrates that exosomal Cx43 plays an important role in exosomal internalization into recipient cells and angiogenesis in hypoxic tumor microenvironment.

**Figure 8:**
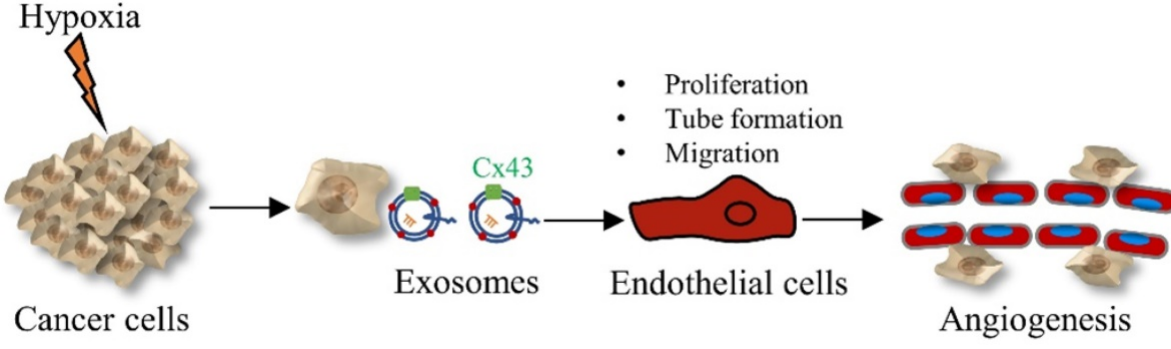
The diagram showing the crucial role of exosomal Cx43 in angiogenesis under hypoxia.

In summary, our data show that exosomes derived from glioma cells under hypoxia promote the proliferation, tube formation and migration of HUVECs through up-regulated exosomal Cx43 (Figure 8). Our findings also provide a new therapeutic strategy for glioma by targeting exosomal Cx43.

## Figures and Tables

**Table 1:** Target sequences of Cx43 shRNA

Name	Sequence
Cx43 shRNA-1	5'-GCTGGTTACTGGTGACAGA-3'
Cx43 shRNA-2	5'-AGAGCACGGCAAGGTGAAA-3'
Cx43 shRNA-3	5'-AGGAAGAGAAGCTAAACAA-3'
Negative control	5'-TTCTCCGAACGTGTCACGT-3'

## Abbreviations

GBM: glioblastoma multiform
HUVECs: human umbilical vein endothelial cells
Exos: exosomes
HIF-1α: hypoxia-inducible factor-1α
Cx43: connexin 43
VEGF: vascular endothelial growth factor
Cxs: connexins
